# What’s in a name?

**DOI:** 10.7554/eLife.32437

**Published:** 2017-10-24

**Authors:** Michael D Schaller, Gary McDowell, André Porter, Dorothy Shippen, Katherine L Friedman, Matthew S Gentry, Tricia R Serio, Wesley I Sundquist

**Affiliations:** 1 Department of BiochemistryWest Virginia UniversityMorgantownUnited States; 2 Future of ResearchAbingtonUnited States; 3 ManylabsSan FranciscoUnited States; 4 Public Affairs OfficeAmerican Society for Biochemistry and Molecular BiologyRockvilleUnited States; 5 Department of Biochemistry and BiophysicsTexas A&M UniversityCollege StationUnited States; 6 Department of Biological SciencesVanderbilt UniversityNashvilleUnited States; 7 Department of Molecular and Cellular BiochemistryUniversity of Kentucky College of MedicineLexingtonUnited States; 8 Department of Biochemistry and Molecular BiologyThe University of Massachusetts, AmherstAmherstUnited States; 9 Department of BiochemistryUniversity of Utah School of MedicineSalt Lake CityUnited States

**Keywords:** point of view, careers in science, postdocs

## Abstract

Numerous concerns have been raised about the sustainability of the biomedical research enterprise in the United States. Improving the postdoctoral training experience is seen as a priority in addressing these concerns, but even identifying who the postdocs are is made difficult by the multitude of different job titles they can carry. Here, we summarize the detrimental effects that current employment structures have on training, compensation and benefits for postdocs, and argue that academic research institutions should standardize the categorization and treatment of postdocs. We also present brief case studies of two institutions that have addressed these challenges and can provide models for other institutions attempting to enhance their postdoctoral workforces and improve the sustainability of the biomedical research enterprise.

## Statement of the problem

Newly minted PhDs frequently continue their research training by working in established laboratories in the US in positions that are often designated as postdocs, but can also be known by more than 30 other names, including Visiting Fellow, Research Fellow or Research Associate (McDowell, 2016). These positions provide training, and contribute intellectual capital and a significant workforce, but an analytical view reveals that the ad hoc expansion of such positions is causing problems.

## Unintended consequences of the problem

Why does the name matter if the job gets done? We argue that a multitude of job titles or designations obscures attempts to address problems in the biomedical research workforce and can also negatively impact individuals in these positions.

If we want to optimize the biomedical research workforce, we need to determine how best to support researchers at each level of their career, including faculty, staff scientists and trainees at all levels. Implementing rational policies that achieve this aim requires us to define the existing workforce, project the composition of the workforce that will be needed in the future, and perform cost/benefit assessments. Although such analyses may seem unnecessary because simple market forces could, in principle, adjust the workforce to meet the needs of the enterprise, biomedical research does not respond to classic market forces in the same way as other industries (Alberts et al., 2014). Therefore, the workforce needs to be managed by other mechanisms. An initial step toward this end is to track the outcomes of postdoctoral training accurately (National Academy of Sciences, National Academy of Engineering, and Institute of Medicine, 2014; Polka et al., 2015; Silva et al., 2016) – a task that has not been performed despite repeated recommendations to do so, and which is made more challenging by difficulties in simply defining the postdoc workforce in the first place.

The true number of postdocs in the US is uncertain, with recent estimates ranging between 30,000 and 80,000 (Heggeness et al., 2016; National Institutes of Health, 2012; Ferguson et al., 2014). For over three decades postdoc numbers have generally increased each year, although the past few years indicate a decline (Garrison et al., 2016). However, dramatic year to year fluctuations in the reported postdoc census at individual institutions, in some cases due to the reclassification of postdocs, contributes uncertainty and makes it difficult to analyze trends (Pickett et al., 2017). Independent academic positions (and equivalent positions in government labs and industry) have grown with a much shallower trajectory than the postdoc population (Schillebeeckx et al., 2013; Larson et al., 2014; Heggeness et al., 2016). Consequently, there is a labor gap in which supply (i.e., the number of postdocs on the job market looking for permanent positions) exceeds demand (the number of positions available; Mason et al., 2016). As a result, highly trained scientists progress through, but then stall in, an ever-lengthening postdoc stage, further increasing the labor gap (Bourne, 2013; Powell, 2015).

Instituting term limits on postdoctoral positions to improve career development and advancement is a recommendation that has emerged from most analyses of the biomedical workforce (Pickett et al., 2015). Unfortunately, this effort has led to the proliferation of new designations for similar positions, which circumvents the goals of the recommendations in several ways. First, scientists in other designations may not receive the training and career development that is provided to their postdoc counterparts. Second, re-designating scientists who have exhausted their postdoc eligibility so that they can simply continue to perform the same work does not constitute advancement. In some cases, postdoc term limits have even had the unintended consequence of pressuring trainees to work without compensation as “volunteers” so that they can better position themselves for career advancement. Consolidation of job titles would allow standardization of training and career development opportunities for all individuals at this career stage.

Other recommendations for sustaining the biomedical research enterprise include increasing postdoc compensation, improving benefits and making postdocs employees of institutions (Pickett et al., 2015; Alberts et al., 2014; National Institutes of Health, 2012; Bourne, 2013). In response to changes to the Fair Labor Standards Act (FLSA) overtime rules proposed in 2016, many US institutions voluntarily increased postdoc salaries. However, the use of non-standard designations has meant that these improved pay scales and benefits packages have not always been extended to researchers who are essentially postdocs. Indeed, only about half of US institutions follow the recommended minimum salary set by the National Institutes of Health (NIH) in their National Research Service Awards ($47,484 in 2017; Ferguson et al., 2014). Standardization of postdoc designations is an important first step toward addressing these discrepancies.

## Solutions to the problem

Institutions have responded to the problem of multiple job designations for postdocs in different ways. Case studies of two such approaches can serve as blueprints for other institutions that wish to standardize their postdoctoral workforces.

In 2004, the Biological Sciences Division at the University of Chicago created two positions for postdocs, Fellows and Scholars, and established policies to ensure equivalent experiences for each (see Table 1). Fellows are paid from grants and fellowships that they themselves bring to the institution, whereas Scholars are paid from funding granted to the institution. Prior to these changes, Fellows were not entitled to benefits from the institution but were required to receive career development training by the terms of their fellowships, whereas Scholars received benefits from the institution but not career development training. As a result of the change in policy, scientists now have equivalent experiences and compensation regardless of their classification as Fellows or Scholars.

**Table 1.** Restructured postdoc designations at the University of Chicago. Postdocs are defined as “Fellows” or “Scholars” depending on the source of their stipend. The appointment process is initiated by the department, but it is then reviewed in the Provost’s Office, the Office of Academic Affairs and the Office of Postdoctoral Affairs to ensure that the terms of the appointment provide appropriate benefits and equitable opportunities for career development. All postdocs are evaluated annually for reappointment to the position, and these requests are reviewed and approved through the same process.

University of Chicago – two postdoc designations – one common experience		
Position	POSTDOCTORAL FELLOW	POSTDOCTORAL SCHOLAR
Stipend	Extramural, e.g. fellowship	Institutional (including from grants)
Benefits	“Supplemental stipend” from institution to provide benefits equivalent to Postdoctoral Scholar	“Special employee” receives benefits from institution
Career Development	Follow criteria outlined in terms of the fellowship	Institutionally mandated to provide similar career development experience

**Figure 1.**
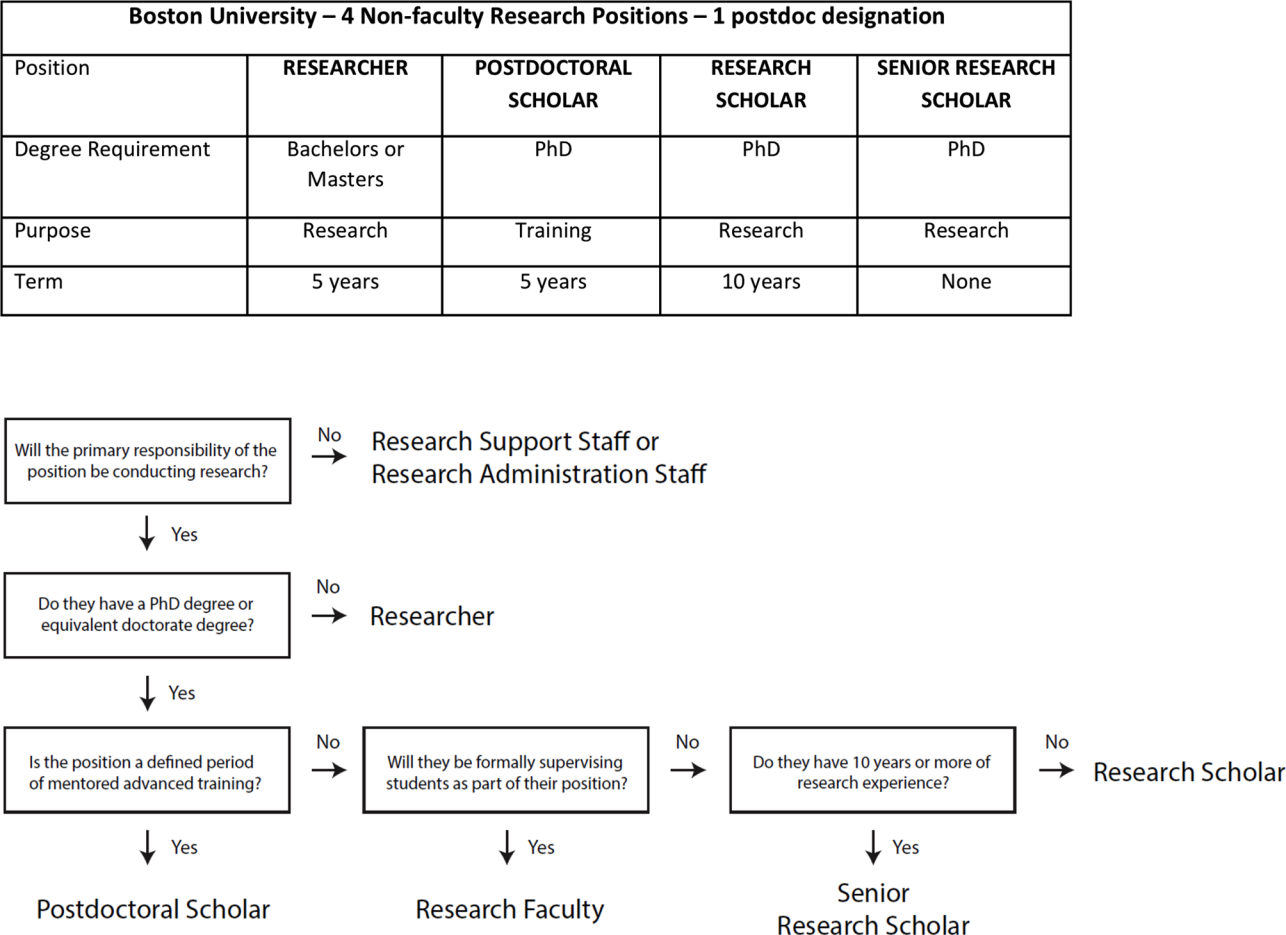
Restructured postdoc designations at Boston University. (**A**) In 2016, a task force considered data on academic research positions nationally and the practices at seven peer institutions, as well as the responsibilities and privileges of the 12 existing non-faculty job titles at Boston University. The resulting recommendations produced four new title positions. One of the positions is Postdoctoral Scholar, which is a training position with a five-year term limit. The other three designations are for researchers with BS or MS degrees (Researcher) and for PhDs who are not in training positions (Research Scholar and Senior Research Scholar – effectively a staff scientist). (**B**) The appropriate job title for a non-faculty position can be determined using a flow chart.

An alternative strategy is to consolidate the different titles into a single designation. In 2016, Boston University charged a task force with streamlining and standardizing all non-faculty research positions, including postdocs. Prior to this effort, positions were classified using a complex job matrix of 12 titles that were applied inconsistently across the campus. The final report recommended consolidating these titles into four distinct non-faculty research positions (see Figure 1). Recommendations for classification were based on degree and experience requirements for the position and whether there was a training component. Boston University is currently in the process of implementing these recommendations.

## How does standardization of job titles overcome impediments?

Standardizing job titles makes it easier to ensure that researchers at a postdoc-equivalent career stage receive equivalent salaries, benefits and career development opportunities. Establishing required salaries for a position not only provides a mechanism for normalizing postdoctoral salaries across an institution, it also provides an opportunity to harmonize salaries with national standards. For example, the Boston University task force recommended setting minimum salaries annually for postdoctoral scholars and other non-faculty research positions, and eliminated unpaid research positions. Similarly, the Biological Sciences Division at Chicago established a required minimum stipend for all postdocs that follows the NIH National Research Service Award scale.

Standardization can also improve career progression; the recommendations of the Boston University task force eliminate designations with overlapping responsibilities and take experience into account, thereby limiting the time before trainees advance into faculty or research staff positions. Even where more than one designation remains in place for similar positions, the benefits and opportunities that researchers receive while in these positions can be standardized. At the University of Chicago, Scholars are “special employees”, and their benefits are subsidized by the University and deducted from payroll. Since Fellows are not employees, Principal Investigators use discretionary funds to contribute to a “supplemental stipend” that covers the costs of benefits, such as health insurance and contributions to a retirement plan. Both Fellows and Scholars are required to have mentoring plans and to participate in programming and events designed to enhance career development.

Standardization of job titles also provides several benefits to the institution. It simplifies tracking, managing and fulfilling reporting requirements about the research workforce at the institution. Further, defining a clear career progression from a postdoctoral position (trainee) to a more advanced research position (staff) at the institution can improve the recruitment and retention of talented researchers.

## Taking action

We have compiled a set of recommendations that other institutions can follow in consolidating their postdoc position designations, based largely upon the successful efforts at Boston and Chicago (see Appendix 1). The recommended consolidation pathway consists of four stages: Assessment, Position Consolidation, Implementation and Monitoring. This guide is intended to help institutions to assess how best to enact postdoc consolidation but it will, of course, be necessary for each institution to tailor these steps to meet their own goals and specific situations.

While there are tangible benefits to simplifying postdoc designations that will positively impact the postdoc experience and sustainability of biomedical research, there may also be conflicting motivations that reduce support for such action. For example, the opportunity to continue a postdoc beyond the term limit (under a different title) may appear to benefit individual postdocs and principal investigators. We argue, however, that it is time to take a broader, longer-term view of the problem and to standardize the postdoc position because individual deviations from this course have contributed to many of the problems discussed above. In short, the biomedical science community has yet to acknowledge and take actions that address many of the concerns surrounding our burgeoning workforce and we believe that standardizing the postdoc position is one such action that all academic and research institutions can and should take.

## Appendix 1

### Recommendations for institutional consolidation of non-faculty postdoctoral positions

We recommend that institutions seeking to consolidate non-faculty postdoctoral research positions consider following the steps outlined below, which are based upon successful consolidation experiences at Boston University and at the University of Chicago. It will, of course, be necessary for each institution to tailor these steps to meet their own goals and specific situations. Consolidation Pathway: Assessment->Position Consolidation->Implementation->Monitoring Step 1: Assessment Non-faculty academic research positions under assessment should be paid and the primary responsibility should be to conduct research. Additional responsibilities such as serving as a principal investigator, supervising students, or teaching may be allowed on a case-by-case basis. Begin by compiling data on all non-faculty academic research positions, subdividing the data into two categories: daily responsibilities and global elements. Examples of day-to-day responsibilities include:

- Training components
- Research experience requirements
- Term limits

Examples of global elements include:

- Compensation (stipend versus salary)
- Eligibility for benefits
- Source of funding
- Visa status
- Discipline

Step 2: Position Consolidation We recommend condensing all non-faculty doctoral research positions into two categories: Postdoctoral Scholar and Research Scholar based on criteria that impact day-to-day responsibilities and are commensurate with research experience and career level. Example: Postdoctoral scholar:

- Requires mentored advanced training
- Does not require previous postdoctoral research experience
- Defined term limit not to exceed 5 years

Research scholar:

- Does not include advanced mentored training
- Requires previous postdoctoral research experience
- Must have fewer than 10 years of previous research experience since PhD completion
- Term limits of 5 years with an option to be renewed once for a maximum of 10 years

Step 3: Implementation Institutional policy creation Develop new university policy to achieve the following:

- Set a minimum salary for consolidated positions;
- Set standard benefits and parity across titles;
- Create renewal restrictions and term limits.

We recommend that the *minimum* salary be set annually and follow a national standard such as the National Institutes of Health’s NRSA scale. This policy will streamline salary determinations and help standardize pay for analogous positions across the institution. However, adoption of an absolute salary scale should be avoided owing to the diversity of disciplines and funding sources, which may have different salary requirements and research field compensation pressures. Position benefits should mirror coverage provided for other university employees, and include:

- a leave policy (vacation/sick/parental)
- a health insurance plan
- a life insurance plan
- a retirement plan (recommended upon promotion to Research Scholar)
- professional development activities
- performance reviews

A supplementary stipend policy should be established to provide funds to cover any benefits that are not supported by certain grants (e.g. health insurance, retirement plan contributions (if provided)), and tax benefits, to ensure equal support for externally and internally funded positions. These funds should be paid by the supervising PI through their source funding. If benefit support is commensurate with established institutional policy, grant dollars received from federal agencies like the National Science Foundation and National Institutes of Health can be used to cover benefits for US citizen and green card holders (PAPPG X.B.1.b; NIHGPS 11.3.10.2). Due to Visa regulations, federal grant funds cannot provide direct support for H1B Visa holders, thus, an institutional plan for covering benefits is required in these cases. Institutions that support postdocs through Ruth L. Kirchstein National Research Awards are not allowed to request additional funds or charge fellows to cover benefits, although fellows can request that the institution deduct benefit costs from his/her stipend. Term limits should be set for each position, with performance and appointments evaluated annually. Annual evaluations should be conducted by the institution’s postdoctoral affairs office or equivalent and human resources, in collaboration with supervising PIs. Strict term and renewal limits should be enforced by human resources. Appointments approaching the end of their terms should be rejected for renewal and the postdoctoral affairs office should work closely with the postdocs to identify career options. The Research Scholar position should have higher salaries and increased benefits (including retirement). These limits will allow retention and promotion of personnel from one position to the next, while facilitating career advancement. All scholars should submit a mentoring/professional development plan annually. These plans should be reviewed and approved by the institution’s postdoctoral affairs office or equivalent, in collaboration with supervising PIs to ensure adherence, progress and effectiveness of each plan. Policy dissemination Human resources should develop an implementation guide that describes how to reassign titles and provides recruitment letter templates. During a specified implementation period, representatives from human resources and the postdoctoral affairs office should conduct town halls and visit departments to inform them about the forthcoming policy changes. Existing appointments should be provided a defined grace period prior to re-designation. Step 4: Monitoring Human resource departments, in concert with the postdoctoral affairs office, should monitor non-faculty doctoral research positions to ensure that new hires and reappointments are in compliance with the newly established policy. The appointment and renewal process for postdoctoral and research scholars should be administered and monitored at the department level, and reviewed by human resources, and/or by the postdoctoral affairs office. Scholar performance and mentoring/professional development plans and progress should be evaluated annually, and scholars evaluated for reappointment (if eligible). Salary/stipend levels should be recalculated during this review process.

## References

[bib1] Alberts B, Kirschner MW, Tilghman S, Varmus H (2014). Rescuing US biomedical research from its systemic flaws. PNAS.

[bib2] Bourne HR (2013). A fair deal for PhD students and postdocs. eLife.

[bib3] Ferguson K, Huang B, Beckman L, Sinche M (2014). National Postdoctoral Association Institutional Policy Report: Supporting and Developing Postdoctoral Scholars.

[bib4] Garrison HH, Justement LB, Gerbi SA (2016). Biomedical science postdocs: an end to the era of expansion. The FASEB Journal.

[bib5] Heggeness MS, Gunsalus KT, Pacas J, McDowell GS (2016). Preparing for the 21st century biomedical research job market: using census data to inform policy and career decision-making – version 1. The Self Journal of Science.

[bib6] Larson RC, Ghaffarzadegan N, Xue Y (2014). Too many Phd graduates or too few academic job openings: The basic reproductive number R0 in academia. Systems Research and Behavioral Science.

[bib7] Mason JL, Johnston E, Berndt S, Segal K, Lei M, Wiest JS (2016). Labor and skills gap analysis of the biomedical research workforce. The FASEB Journal.

[bib8] McDowell G (2016). Four reasons we don’t need 37 names for postdocs. ASBMB Today.

[bib9] National Academy of Sciences, National Academy of Engineering, and Institute of Medicine (2014). The Postdoctoral Experience Revisited.

[bib10] National Institutes of Health (2012). Biomedical Research Workforce Working Group Report.

[bib11] Pickett CL, Corb BW, Matthews CR, Sundquist WI, Berg JM (2015). Toward a sustainable biomedical research enterprise: Finding consensus and implementing recommendations. PNAS.

[bib12] Pickett C, Bankston A, McDowell GS (2017). The GSS is an unreliable indicator of biological sciences postdoc population trends. bioRxiv.

[bib13] Polka JK, Krukenberg KA, McDowell GS (2015). A call for transparency in tracking student and postdoc career outcomes. Molecular Biology of the Cell.

[bib14] Powell K (2015). The future of the postdoc. Nature.

[bib15] Schillebeeckx M, Maricque B, Lewis C (2013). The missing piece to changing the university culture. Nature Biotechnology.

[bib16] Silva EA, Des Jarlais C, Lindstaedt B, Rotman E, Watkins ES (2016). Tracking career outcomes for postdoctoral scholars: A call to action. PLOS Biology.

